# Improved glucose metabolism by *Eragrostis tef* potentially through beige adipocyte formation and attenuating adipose tissue inflammation

**DOI:** 10.1371/journal.pone.0201661

**Published:** 2018-08-02

**Authors:** Mengistu Lemecha, Katsutaro Morino, Daniel Seifu, Takeshi Imamura, Fumiyuki Nakagawa, Aki Nagata, Takuya Okamato, Osamu Sekine, Satoshi Ugi, Hiroshi Maegawa

**Affiliations:** 1 Division of Endocrinology and Metabolism, Department of Medicine, Shiga University of Medical Science, Otsu, Japan; 2 School of Medicine, Department of Biochemistry, Addis Ababa University, Addis Ababa, Ethiopia; 3 Division of Molecular Pharmacology, Faculty of Medicine, Tottori University, Yonago, Japan; 4 CMIC Pharma Science, Osaka, Japan; Tohoku University, JAPAN

## Abstract

**Background:**

Teff is a staple food in Ethiopia that is rich in dietary fiber. Although gaining popularity in Western countries because it is gluten-free, the effects of teff on glucose metabolism remain unknown.

**Aim:**

To evaluate the effects of teff on body weight and glucose metabolism compared with an isocaloric diet containing wheat.

**Results:**

Mice fed teff weighed approximately 13% less than mice fed wheat (*p* < 0.05). The teff-based diet improved glucose tolerance compared with the wheat group with normal chow but not with a high-fat diet. Reduced adipose inflammation characterized by lower expression of *TNFα*, *Mcp1*, and *CD11c*, together with higher levels of cecal short chain fatty acids such as acetate, compared with the control diet containing wheat after 14 weeks of dietary treatment. In addition, beige adipocyte formation, characterized by increased expression of *Ucp-1* (~7-fold) and *Cidea* (~3-fold), was observed in the teff groups compared with the wheat group. Moreover, a body-weight matched experiment revealed that teff improved glucose tolerance in a manner independent of body weight reduction after 6 weeks of dietary treatment. Enhanced beige adipocyte formation without improved adipose inflammation in a body-weight matched experiment suggests that the improved glucose metabolism was a consequence of beige adipocyte formation, but not solely through adipose inflammation. However, these differences between teff- and wheat-containing diets were not observed in the high-fat diet group.

**Conclusions:**

Teff improved glucose tolerance likely by promoting beige adipocyte formation and improved adipose inflammation.

## Introduction

Teff (*Eragrostis tef*) is an ancient, indigenous cereal crop and the main staple food in Ethiopia that is believed to have been domesticated before 1000–4000 BCE [1]. It is also grown in India, Europe, Australia and the United States of America [2], and has become popular because it is gluten-free [3]. Teff is one of the world’s smallest grains and is difficult to refine, making its flour rich in fiber from the bran and germ [4]. Its name is derived from the Amharic term “tefa”, meaning “lost”, because of its tiny size. It is estimated that 20%–40% of the carbohydrates in teff are resistant starches [5].

Recently, studies have shown that dietary fiber plays a role in lowering inflammation [6]. Dietary fiber also reduces body weight by acting on satiety mechanisms and post-prandial glycemia by inhibiting absorption [7]. In addition, diets rich in whole grains and resistant starches improve insulin responsiveness and reduce the incidence of type 2 diabetes compared with diets based on refined grains [8].

Adipose tissue inflammation is a metabolic disorder implicated in the development of insulin resistance mediated by macrophage recruitment [9]. Pro-inflammatory cytokines released from adipose tissue consequently contribute to the progression of type 2 diabetes mellitus. Adipose tissue inflammation is characterized by the increased presence of crown-like structures and upregulation of proinflammatory genes such as tumor necrosis factor α (*TNFα*) and monocyte chemoattractant protein 1 (*Mcp1*; *CCL2*), and the macrophage marker, *F4/80* [10].

Short chain fatty acids (SCFAs), such as acetate, propionate, and butyrate, are products of the microbiota in the intestine. Resistant starches and dietary fibers are the main substrates of microbiota that produce SCFAs [11]. Notably, acetate is the most dominant product in the intestine and attenuates chronic inflammation through activation of regulatory T cells [12]. Increasing evidence shows that SCFAs are potential targets for preventing or counteracting obesity and its associated disorders, such as dysregulated glucose metabolism and insulin resistance [13]. Although teff has become a familiar health food because of its gluten-free characteristic, its effect on glucose metabolism remains completely unknown. Therefore, we evaluated the effect of teff on glucose metabolism *in vivo* in mice.

## Materials and methods

### Animals and dietary groups

Male C57BL/6J mice were housed with a 12-h light–dark cycle at 24°C. Animals had free access to food and water. At 8 weeks, mice were randomly divided into four groups: (1) chow diet plus 30% (w/w) wheat (CD-wheat), (2) chow diet plus 30% (w/w) teff (CD-teff), (3) high-fat diet plus 30% (w/w) wheat (HFD-wheat), and (4) high-fat diet plus 30% (w/w) teff (HFD-teff). Daily food intake was estimated by subtracting the food weight each day from the initial food weight of the previous day. Mice were fed with the study diet from 8 to 22 weeks of age. All experimental protocols were approved by the Animal Care and Use Committee of Shiga University of Medical Science (Identification code: 2016-2-6, Approval date: 2016-03-07). Animals were treated in accordance with the Guidelines of the United States National Institutes of Health. All surgeries were performed under sevoflurane anesthesia and efforts were made to minimize suffering throughout the study.

### Tissue collection

Mice were euthanized after 5 h of fasting by intraperitoneal administration of 10% pentobarbital with sevoflurane inhalation before immediate tissue collection. Inguinal white adipose tissue (iWAT) and epididymal WAT (eWAT) were dissected immediately, snap frozen in liquid nitrogen, and stored at −80°C until analysis, except for histological analysis. Ileum and cecum feces were collected from the mice under deep anesthesia. Feces were collected and stored at −80°C until further analysis.

### Diet composition

Teff (*Eragrostis tef*) was provided by The Teff Company (Nampa, ID, USA). Wheat flour was from Nisshin Flour (Tokyo, Japan). The composition and energy density of experimental diets are shown in Table 1. The control chow corresponded to the standard rodent diet used by the American Institute of Nutrition (AIN-93G diet) from Oriental Yeast Co., Ltd. (Tokyo, Japan). The amounts of micronutrients (minerals and vitamins) added were based on recommendations from the AIN-93G reference diet were adapted to the respective contents of each diet. The high fat diet was purchased from Dyets Inc. [27% w/w safflower oil, 59% fat-derived calories; #112245, Bethlehem, PA, USA].

**Table 1:** Composition and energy density of experimental diets.

Variety	Teff (Ivory) (Per 100 g or %)	Wheat (Per 100 g or %)
Calories, Kcal	369	366
Total Fat, g	3.1	1.5
Saturated Fat, g	0.8	0.34
Total Carbohydrate, g	74.5	75.9
Dietary Fiber, g	10.7	2.5
Starch, g	63.8	73.4
Protein, g	10.8	8.1
Ash, g	2.4	0.33
Moisture, %	9.2	14.17
Vitamin A, %	NT^a^	0
Vitamin C, %	NT^a^	0
Calcium, mg	121	20
Iron, mg	10.3	0.5

^a^Not tested because of insufficient levels

### In vitro fermentation

Teff and wheat fermentation was performed according to a previous report [14]. Briefly, 20 g of teff or wheat flour were mixed with 37 mL of distilled water in a 250 mL beaker. The contents of the beakers were thoroughly mixed after addition of 3 mL (5%) of a previously fermented batch (called *ersho* by the local people) to the fermenting substrate as a starter culture. Fermentation was performed at room temperature (23 ± 1°C) for 72 h. Samples were stored at –80°C until further analysis.

### Glucose and insulin tolerance tests

The glucose and insulin tolerance tests (GTT and ITT, respectively) were performed at the indicated time points. For the oral glucose tolerance test (OGTT), glucose (2 g/kg body weight) was administered to mice by gavage after 15 h of fasting. For the intraperitoneal glucose tolerance test (IPGTT), mice received glucose (2 g/kg body weight) by intraperitoneal injection after 5 h of fasting. For the ITT, mice received insulin by intraperitoneal injection (0.5 units/kg body weight; human insulin, Eli Lilly, Inc., Indianapolis, IN, USA) after 5 h of fasting. Blood glucose levels were measured at 0, 15, 30, 60, 90, and 120 min after insulin administration.

### Meal tolerance test

To estimate postprandial glucose excursion in each group, the total mixed meal tolerance test (OMTT) was performed after 6 weeks of dietary treatment by oral gavage of the assigned diet (2.2 g/kg body weight, 33% solution in dH_2_O), after 15 h of fasting.

### Body temperature measurements

For rectal temperature measurements, mice were placed in individual cages at room temperature (22–25°C). Rectal temperature was measured by a rectal probe of a digital laboratory thermometer (RET-3-ISO, type T thermocouple; Physitemp Instruments Inc, Clifton, NJ, USA). The lubricated probe was inserted ~1.5 cm into the rectum for ~30 sec prior to each recording.

### Blood analysis

Blood glucose levels were measured using whole blood taken from the tail vein using a Glutest sensor (Sanwa Kagaku, Nagoya, Japan). Blood samples were collected from the tail vein with a heparinized tube and centrifuged at 700 × *g* for 15 min. Plasma samples were stored at– 80°C until further analysis. Plasma levels of insulin were determined using a mouse insulin ELISA kit (Morinaga, Kanagawa, Japan). For measuring mouse glucagon like peptide 1 (GLP1), fasting plasma samples were stabilized with a protease inhibitor cocktail (Complete; Roche, Mannheim, Germany) and a specific dipeptidyl peptidase IV inhibitor (EMD Millipore Corp, St.Charles, Missouri, USA). GLP1 plasma levels were determined using a mouse GLP1 ELISA kit (LSBio, Seattle, WA, USA). For blood chemistry analysis blood sample were collected from mice fed with CD-wheat, CD-teff, HFD-wheat or HFD-teff for 9 weeks, after 15 h of fasting or feeding under deep anesthesia. The plasma levels of calcium and inorganic phosphate (enzymatic method), and iron [direct colorimetric method (Nitroso-PSAP)] were measured by the Nagahama Life-science Laboratory of Oriental Yeast Co., Ltd. (Nagahama, Shiga, Japan).

### SCFA analysis

SCFAs in the cecum and *in vitro* fermentation experiments were performed by LC-MS/MS. For SCFAs extraction (acetate, propionate, n-butyrate, and n-valerate) from samples, ethanol:water (3:7, v/v) was added at room temperature. After extraction, internal standard solutions, 2-nitrophenyl hydrazine, and condensation reagent were added to the tube for derivatization. Test tubes were placed in ice water for 60 min for the labeling reaction. Alkaline solution was added to stop the reaction and tubes were left in ice water for 30 min. After the reaction stopped, an acidic solution and hexane were added for liquid-liquid extraction. The hexane layer was removed, and then ether was added for liquid-liquid extraction. The ether layer was transferred to another test tube and dried under a nitrogen stream. Ammonium formate/methanol solution was added to re-fuse the residues in the tubes and an aliquot was injected into the LC-MS/MS system. Liquid chromatography was performed using an ACQUITY UPLC system (Waters, Milford, MA, USA), separated using an analytical column (AQUITY HSS T3 2.1 × 150 mm, 1.8 μm; Waters). For the detector, an API4000 tandem mass spectrometer (AB Sciex, Foster City, CA, USA) was used.

### Histology, immunofluorescence and immunohistochemistry

For histological examination, a portion of the adipose tissue (inguinal fat and epididymal fat) was fixed with 3.7% neutrally buffered formaldehyde and embedded in paraffin. Hematoxylin and eosin staining was carried out on paraffin sections using standard methods [15]. For immunostaining, the paraffin-embedded sections were deparaffinized and incubated for 30 min with 0.3% H_2_O_2_ in methanol to block endogenous peroxidase. Subsequently, endogenous avidin and biotin were blocked using an Avidin-Biotin Blocking kit (DAKO, Carpentaria, CA, USA), as described previously [16]. Next, either immunofluorescence or immunohistochemical staining was conducted. Immunofluorescence staining: tissue sections were then incubated overnight at 4°C with a 1:50 dilution of primary antibodies (anti-F4/80, MCA497, Bio-Rad, Hercules, CA, USA; anti-TNFα, ab6671, Abcam, Cambridge, MA, USA; anti-Perilipin, 9349S, Cell Signalling Technology, Danvers, MA, USA). Sections were then incubated with a 1:1000 dilution of secondary antibodies for 1h at room temperature [rhodamine-conjugated anti-rat secondary antibody (Cappel, MP Biomedicals, LLC, Solon, OH, USA) for F4/80 and FITC-conjugated anti-rabbit (Cappel, MP Biomedicals, LLC) for TNFα and Perilipin)]. Nuclei were stained with DAPI. Tissue slides were mounted with vectashield mounting media (Vector Laboratories, Burlingame, CA, USA) and images were obtained using an Olympus FLUOVIEW FV1000 confocal laser scanning microscope with an oil-immersion objective lens (Olympus Corp, Tokyo, Japan). Double-color immunofluorescence analysis was performed to detect F4/80 and TNFα positive cells as described previously [17].

Immunohistochemical staining: tissue sections were incubated overnight at 4°C with primary antibodies (anti-F4/80, MCA497, Bio-Rad; anti-Ucp-1, U6382, Sigma, St. Louis, MO, USA), followed by 1 h incubation at room temperature with peroxidase-conjugated anti-rabbit IgG secondary antibodies [Histofine Simple Stain Max-PO (R); Nichirei, Tokyo, Japan] at a 1:1000 dilution. Immune complexes were visualized using the peroxidase stain DAB kit (brown stain; Nacalai Tesque, Kyoto, Japan) according to the manufacturer’s instructions. Confocal microscopy imaging was used to visualize the results.

### Adipocyte size and counting

To perform adipocyte number and size analyses, we used semi-automated morphometry with immunofluorescence staining of paraffin sections of inguinal WAT pads using perilipin antibody [18]. Briefly, iWAT pads of mice from each group were fixed in formalin and embedded in paraffin, followed by immunofluorescence staining against perilipin as stated above. Adipocyte size and number per area were measured per mouse from randomly selected fields using semi-automated morphometry (ImageJ, plugin Adipocytes Tool; National Institutes of Health, Bethesda, MD, USA; http://imagej.nih.gov/ij/). Measurements were obtained from three individual animals per group.

### Villus measurements

Villus length was measured using image J software (http://rsb.info.nih.gov/ij/) according to a previous report [19]. For all determinations at least five villi per slide were analyzed by an independent investigator who was blinded to the treatment.

### Pair-feeding protocol

Weight matching was conducted by calorie restriction [20, 21]. Briefly, male, C57Bl/6 mice were either subjected to calorie restriction (CR) (wheat group, n = 3), or fed *ad libitum* (Teff group, n = 4). CR was initiated at 10 weeks of age with 5% CR for one week followed by 10% CR for 5 weeks. Food intake was typically adjusted every three days.

### Real-time (RT) qPCR analysis

Total RNA from frozen adipose tissue was extracted using the RNeasy lipid tissue mini kit (Qiagen, Germantown, MD, USA) and analyzed using a Nanodrop spectrophotometer. The qPCR reaction setup and plate preparation were standardized and carried out according to standard operating protocols provided by the manufacturers. Single-stranded cDNA was synthesized from 1.5 μg of total RNA using the Prime Script RT Reagent Kit (Takara Bio, Shiga, Japan), and endogenous genomic DNA was degraded with DNase I (Life Technologies, Carlsbad, CA, USA). The primers used are shown in Table 2. RT qPCR experiments were carried out with SYBR Green PCR master mix (Life Technologies) using an ABI 7500 Fast RT PCR System (Applied Biosystems, Foster City, CA, USA). All quantitative data were normalized against the expression levels of *36B4*. RT qPCR conditions were 95°C for 10 min, followed by 40 cycles of 95°C for 15 s and 60°C for 1 min.

**Table 2:** RT qPCR primer sequences.

	Forward primer	Reverse primer
*36B4* [22]	5`-GCCGTGATGCCCAGGAAGA-3`	5-`CATCTGCTTGGAGCCCACGTT-3`
*TNFα* [23]	5`-CCCTCACACTCAGATCATCTTC-3`	5`-GCTACGACGACGTGGGCTACAG-3`
*MCP1* [24]	5`-GCCCCACTCACCTGCTGCTACT-3`	5`-CCTGCTGCTGGTGATCCTCTTGT-3`
*F4/80* [24]	5`-CTTTGGCTATGGGCTTCCAGTC-3`	5`-GCAAGGAGGACAGAGTTTATCGT-3`
*CD11c* [23]	5`-CTGGATACCCTTTCTTCTGCTG-3`	5`-CCACACTGTGTCCCAACTC-3`
*Adiponectin* [25]	5'-GGCAGGAAAGGAGAACCTGG-3'	5'-AGCCTTGTCCTTCTTGAAGA-3'
*Ucp-1* [22]	5'-GGGCATTCAGAGGCAAATCAGCTT-3`	5'-ACACTGCCACACCTCCAGTCATTA-3`
*Cidea* [22]	5'-ACTTCCTCGGCTGTCTCAATGTCA-3`	5'-TCAGCAGATTCCTTAACACGGCCT-3`
*PGC1-α* [26]	5'-CATTTGATGCACTGACAGATGGA-3`	5'-CCGTCAGGCATGGAGGAA-3`
*Tfam* [27]	5'-GCAGCCCTGTGGAGGGAGCTA-3`	5'-TCTGCCGGGCCTCCTTCTCC-3`
*Lcad* [28]	5'- AAACGTCTGGACTCCGGTTC-3`	5'-GTACCACCGTAGATCGGCTG-3`
*Mcad* [29]	5'-TCAAGATCGCAATGGGTGCT-3`	5'-GCTCCACTAGCAGCTTTCCA-3`

### Statistical analysis

Data are expressed as mean ± SE. The level of statistical significance was determined using Student's two-tailed *t*-test when the difference between the means of two populations was considered. For differences using multiple comparisons, a one-way ANOVA followed by Tukey's *post hoc* test was performed. *p* < 0.05 was considered statistically significant.

## Results

### Teff stabilizes weight gain with a normal diet

Compared with wheat, teff contains comparable calories and total carbohydrates, but is lower in starch, and higher in dietary fiber, fat, protein, and ash compared with wheat (Table 1).

**Fig 1:**
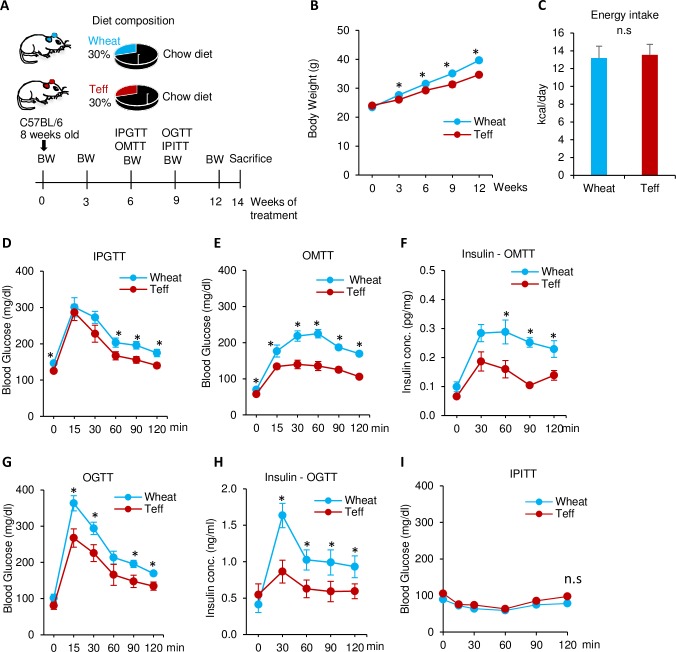
Comparison of body weight and glucose metabolism between mice fed a chow diet with wheat (CD-wheat: Blue) or teff (CD-teff: Red). A: Study design. B: Body weight. C: Energy intake. D: Intraperitoneal glucose tolerance test (IPGTT) at week 6 (2.0 g/kg). E: Blood glucose levels after oral mixed meal administration of each assigned diet (2.2g/kg body weight, 33% solution in dH_2_O) after 16 h of fasting at week 6. F: Plasma insulin levels during OMTT. G: Oral glucose tolerance test (OGTT) at week 9 (2 g/kg). H: Insulin concentration during OGTT. I: Intraperitoneal insulin tolerance test (IPITT) at week 9 (0.5 U/kg). * *p* < 0.05, n.s. = not significant. n = 5–9 in each groups.

Mice were separated into two groups: chow diet with teff (CD-teff) or chow diet with wheat (CD-wheat, 30% w/w; Fig 1A). The CD-teff group showed a significantly lower rate of weight gain compared with the CD-wheat group (Fig 1B). At the end of the feeding period, mice fed teff weighed almost 12.5% less (*p* < 0.05). However, there were no significant differences in food intake observed between wheat- and teff-fed mice (Fig 1C).

### Teff diet improves glucose tolerance in mice

GTT and ITT were performed to evaluate the effects of teff on glucose metabolism. Mice fed the teff-based diet showed significantly lower plasma glucose levels during the IPGTT at 60, 90, and 120 min after the glucose load after 6 weeks of treatment (Fig 1D). To estimate the postprandial levels of glucose and insulin, OMTT was performed. During the OMTT, glucose levels were significantly lower in the teff group at all time points after 6 weeks of treatment (*p* < 0.05) (Fig 1E). Insulin levels were also significantly lower in the teff group during the OMTT at 60, 90, and 120 min (Fig 1F). The blood glucose levels and plasma insulin levels during the OGTT after 9 weeks of treatment were significantly lower in the CD-teff group compared with the CD-wheat group (*p* < 0.05) (Fig 1G and 1H, respectively). However, the intraperitoneal insulin tolerance test (IPITT) after 9 weeks of treatment showed no significant differences in blood glucose levels after insulin injection (Fig 1I).

### Teff diet improves insulin sensitivity in the HFD model

**Fig 2:**
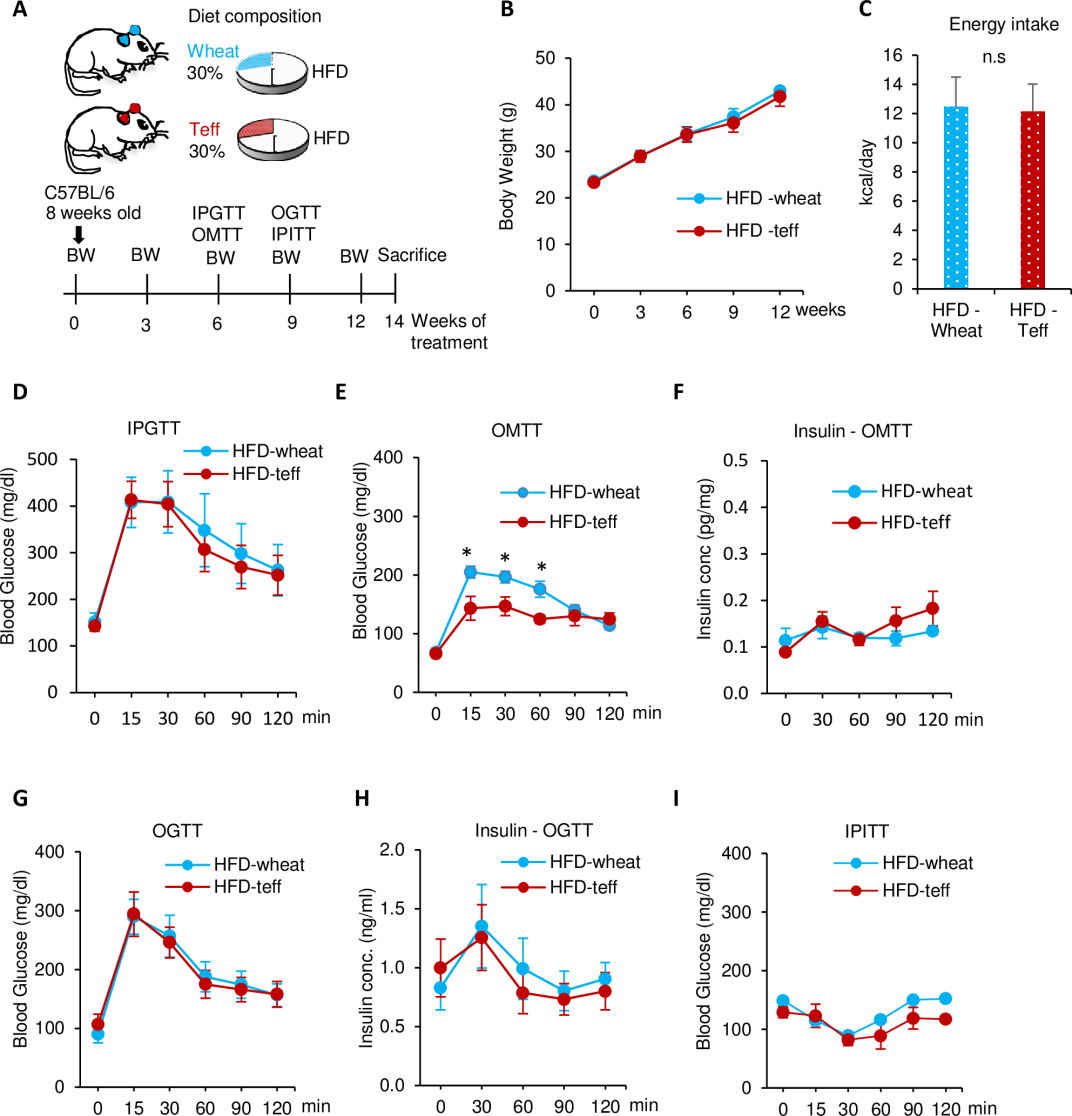
Comparison of body weight and glucose metabolism between mice fed a high-fat diet with wheat (HFD-wheat: Blue) or teff (HFD-teff: Red). A: Study design. B: Body weight. C: Energy intake. D: Intraperitoneal glucose tolerance test (IPGTT) at week 6 (2 g/kg). E: Blood glucose levels after oral mixed meal administration of each assigned diet (2.2g/kg body weight, 33% solution in dH_2_O) after 16 h of fasting at week 6. F: Plasma insulin levels during OMTT. G: Oral glucose tolerance test (OGTT) at week 9 (2 g/kg). H: Insulin concentration during OGTT. I: Intraperitoneal insulin tolerance test (IPITT) at week 9 (0.5 U/kg). * *p* < 0.05, n.s. = not significant. n = 5–9 in each groups.

Mice were separated into two groups and fed either the HFD-wheat or HFD-teff diet (Fig 2A). There was no significant difference in body weight and food intake between the two groups (Fig 2B and 2C respectively). During the OMTT, the glucose levels were significantly lower in the teff group compared with the wheat group at 15, 30 and 60 min after oral meal administration, after 6 weeks of treatment (*p* < 0.05) (Fig 2E). However, the insulin levels in the OMTT were comparable between the two groups (Fig 2F). Furthermore, in the IPGTT, OGTT, and IPITT, mice fed the HFD-teff diet displayed no significant differences in glucose or insulin levels compared with the HFD-wheat group (Fig 2D, 2G and 2I).

### Teff diet reduces adipose inflammation with a normal diet

**Fig 3:**
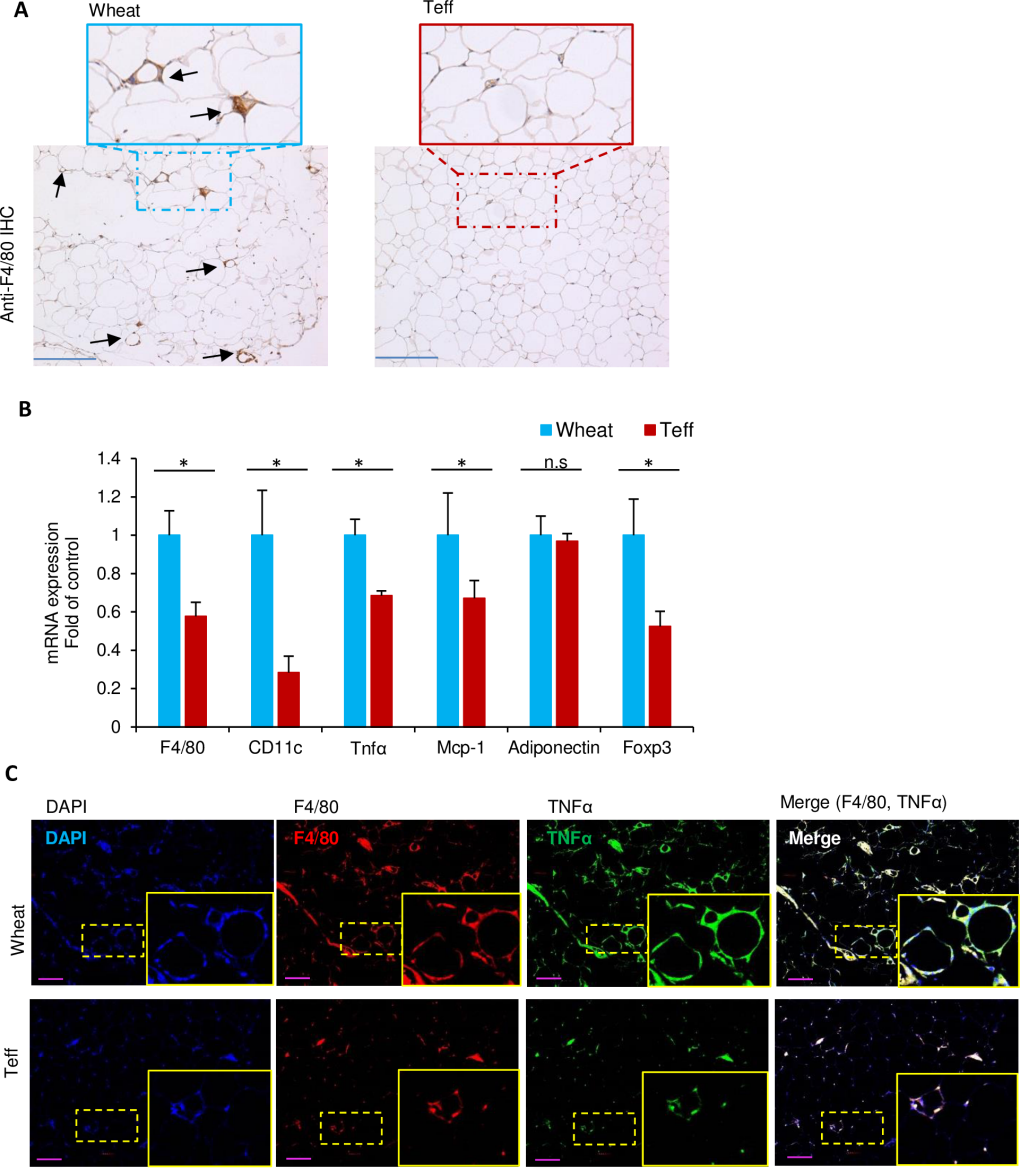
Adipose tissue inflammation in mice fed a chow diet with wheat (CD-wheat) or chow diet with teff (CD-teff) for 14 weeks. A: Immunostaining of the macrophage marker F4/80 (brown) in adipose tissue (scale bar = 100 μM). B: mRNA levels of the macrophage marker, *F4/80* and *CD11c*, tumor necrosis factor α (*TNF*α), monocyte chemoattractant protein-1 (*Mcp-1*), Forkhead Boxprotein P3 *(Foxp3*), and adiponectin in adipose tissue. All mRNA expression data were normalized to *36B4*. C: Immunofluorescence staining for DAPI (blue), F4/80 (red) and TNFα (green) in adipose tissue (scale bar, 100 μm). * *p* < 0.05. n.s. = not significant.

To further investigate the effect of a teff-based diet on glucose metabolism, we next examined the effects of a teff diet on adipose inflammation in the epididymal fat pad after 14 weeks of dietary treatment. As shown in Fig 3A, the CD-wheat group exhibited higher macrophage infiltration compared with the CD-teff group, as determined by F4/80 staining, which is a macrophage marker. Surprisingly, the crown-like structures were not detected in the teff diet mice (Fig 3A). Furthermore, the gene expression of proinflammatory cytokines such as *Mcp1* and *TNF*α, in the adipose tissue was significantly lower in the CD-teff group (*p* < 0.05) along with the lower expression of macrophage markers (Fig 3B). This was confirmed by immunofluorescence in which the merged staining of F4/80 and TNFα revealed reduced adipose inflammation in the CD-teff group compared with the CD-wheat group (Fig 3C). The expression of *Foxp3*, a marker of regulatory T cells, was reduced in the CD-teff group compared with the CD-wheat group (Fig 3B).

### Teff does not ameliorate adipose inflammation in the HFD model

**Fig 4:**
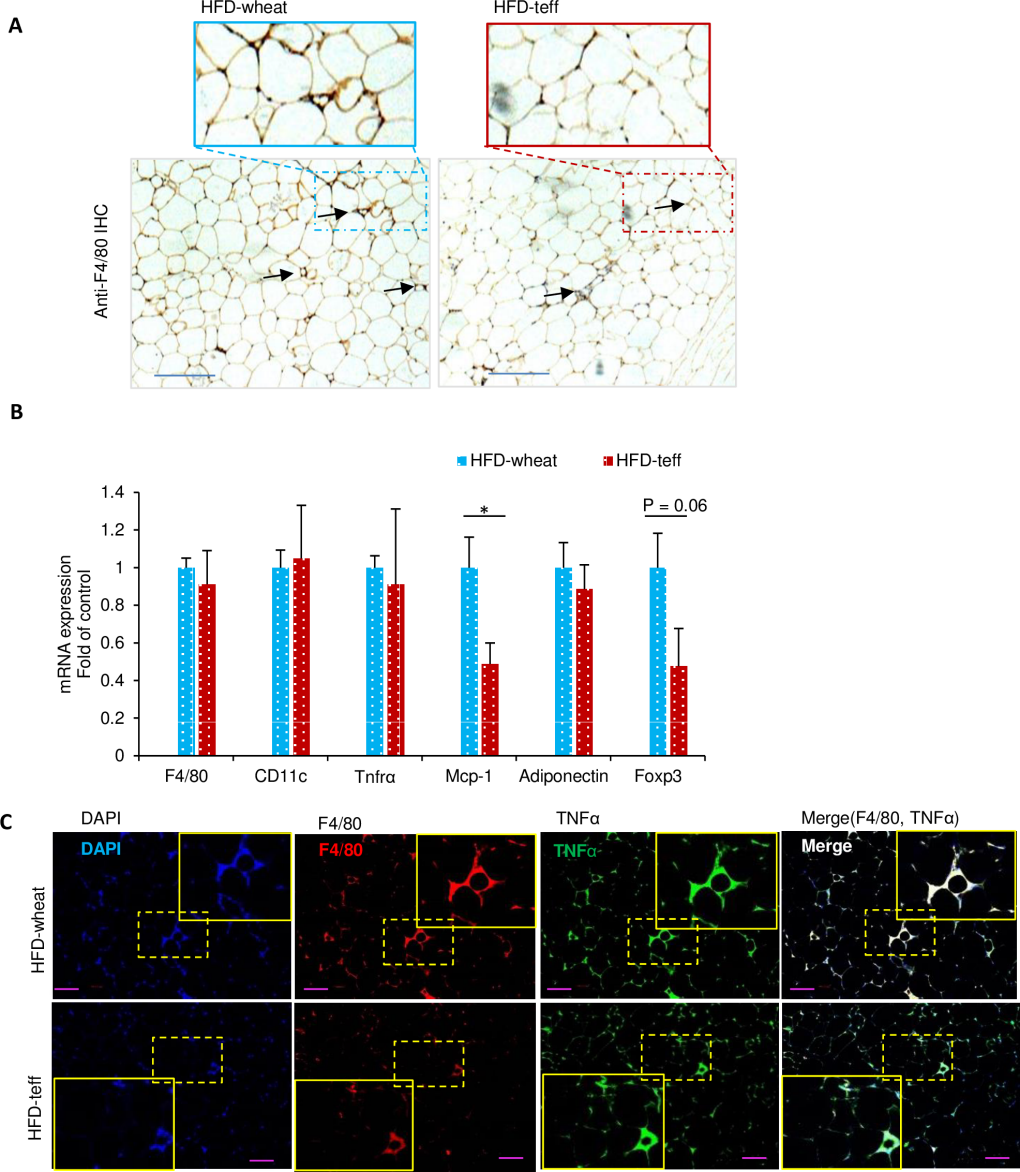
Adipose tissue inflammation in mice fed a high-fat diet with wheat (HFD-wheat) or high-fat diet with teff (HFD-teff). A: Immunostaining of the macrophage marker F4/80 (brown) of adipose tissue (scale bar = 100 μM). B: mRNA levels of macrophage marker, *F4/80* and *CD11c*, tumor necrosis factor (*TNFα*), monocyte chemoattractant protein-1 (*Mcp1*), Forkhead Boxprotein P3 *(Foxp3*), and adiponectin in adipose tissue. All mRNA expression data were normalized to *36B4*. C: Immunofluorescence staining with DAPI (blue), and antibodies for F4/80 (red), and TNFα (green) in adipose tissue (scale bar, 100 μm). * *p* < 0.05. n.s. = not significant.

Immunohistochemical staining against F4/80 showed comparable results between the HFD-teff and HFD-wheat groups after 14 weeks of dietary treatment (Fig 4A). Consistent with the histological findings, there were no significant differences in the gene expression of proinflammatory cytokines between the two groups (Fig 4B), except for lower expression of *Mcp1* in the HFD-teff group compared with the HFD-wheat group (Fig 4B). This was confirmed by immunofluorescence staining of F4/80 and TNFα demonstrating comparable adipose inflammation between the HFD-teff and HFD-wheat groups (Fig 4C).

### SCFAs concentrations in the cecum

**Fig 5:**
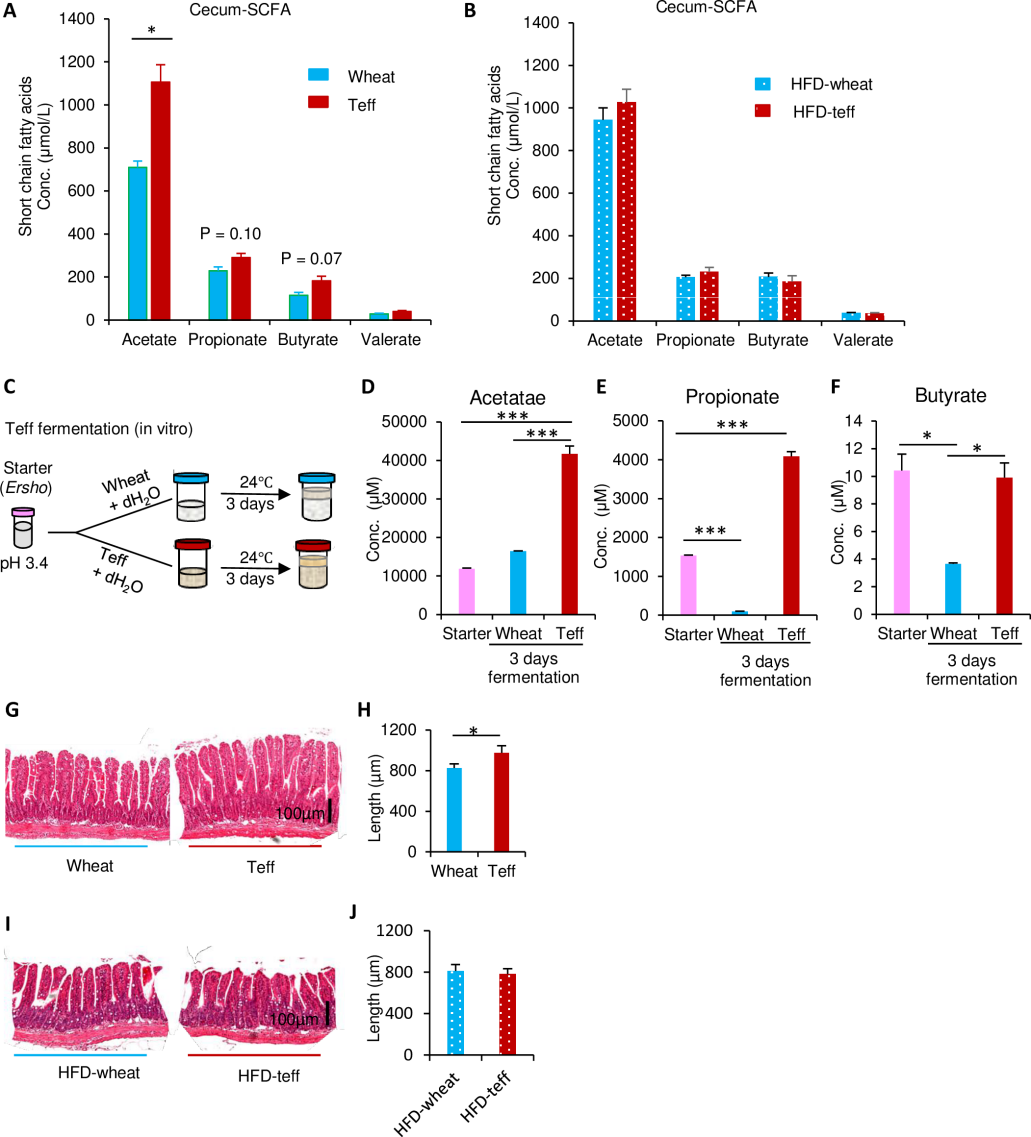
Cecal short chain fatty acids (SCFAs) concentrations. A: Cecal SCFAs concentrations after 15-h fasting by LC-MS in mice fed with CD-wheat or CD-teff for 14 weeks. B: Cecal SCFAs concentrations were measured by LC-MS after 15-h fasting in mice fed with HFD-wheat or HFD-teff for 14 weeks. C: *In vitro* teff and wheat fermentation methods. D–F: Acetate, propionate and buyrate concentrations SCFA extracted from *in vitro* fermented teff and wheat by LC-MS. G& I: Hematoxylin and eosin stained slides of villi. H and J: Ileum villus length determined as indicated in the Materials and Methods for the CD-teff group compared (right panel) with the CD-wheat group (left panel). n = 3–4. *p < 0.05, *** *p* < 0.0001. n.s. = not significant.

SCFAs are products from the metabolism of dietary fiber by microbiota. Thus, the concentrations of major SCFAs in the cecum after 14 weeks of dietary treatment were evaluated (Fig 5A). Acetate levels were dramatically higher in the CD-teff group compared with the CD-wheat group (1105 ± 80 vs. 709 ± 30 μmol/L, *p* < 0.05). Furthermore, the concentrations of propionate, butyrate, and valerate tended to be higher in the CD-teff group compared with the CD-wheat group. In contrast, there were no significant differences in the SCFAs concentrations between the HFD-wheat and HFD-teff groups (Fig 5B). To determine the source of SCFAs, *in vitro* fermentation was performed with the starter that is commonly used in the preparation for cooking injera in Ethiopia (Fig 5C). The concentration of acetate and propionate was dramatically increased in the *in vitro* fermented teff but butyrate was unchanged compared with the starter. In contrast the concentration of propionate and butyrate decreased in the *in vitro* fermented wheat compared with the starter (Fig 5D–5F). SCFAs are an important energy substrate for intestinal epithelium. The villi height in the ileum was higher in the CD-Teff group than in the CD-wheat group, suggesting a role for SCFAs, which were abundant in the cecum of mice from the CD-Teff group (Fig 5G and 5H). However, this difference in villus size was not observed in the HFD-teff group compared with the HFD-wheat group (Fig 5I and 5J).

### Beige adipocyte formation in the teff diet groups

**Fig 6:**
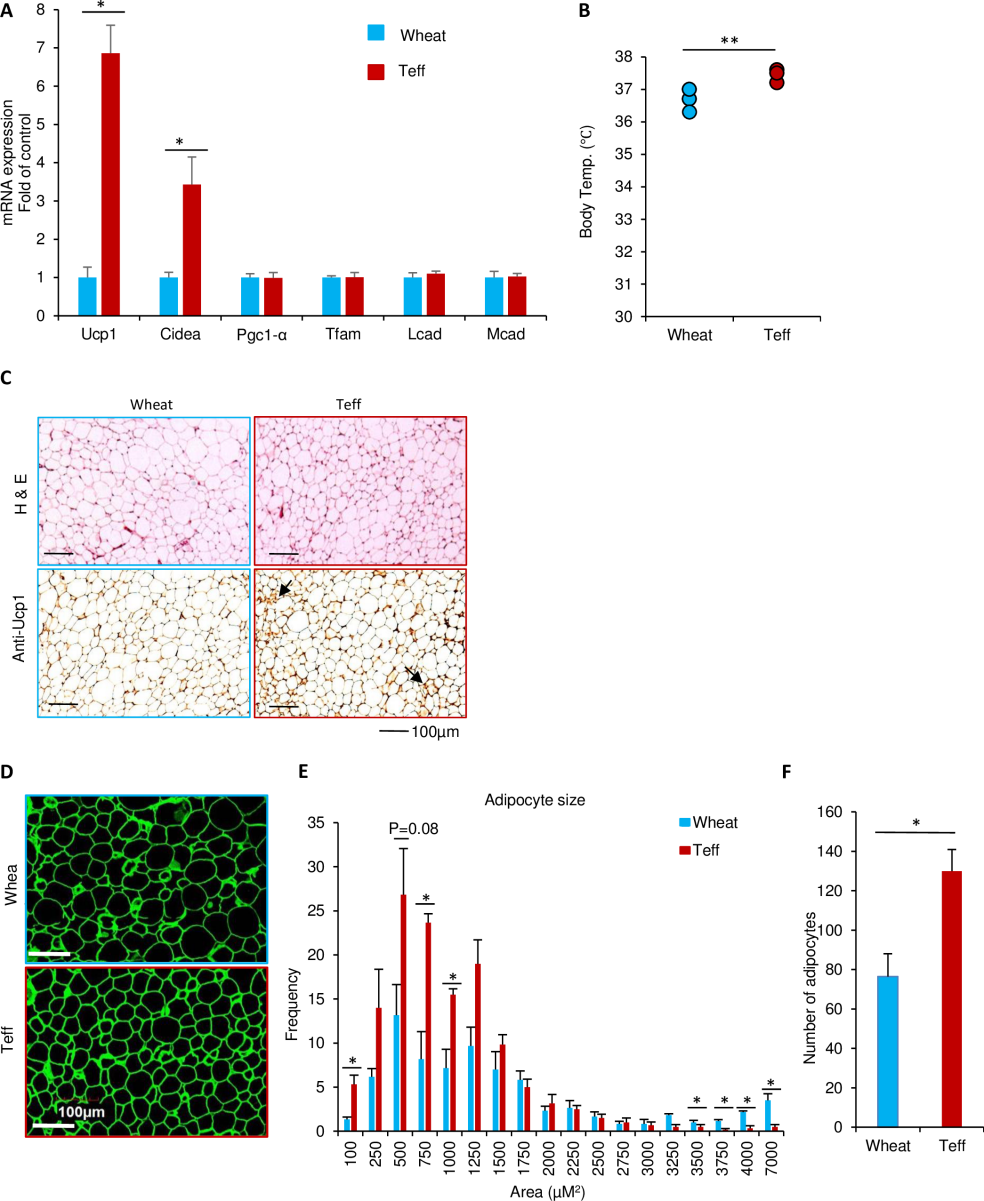
The possible role of beige adipocyte formation in CD-teff treated mice. A: mRNA levels of thermogenic and beige adipocyte marker genes in the inguinal adipose tissue from mice fed for 14 weeks with CD-what or CD-teff. All mRNA expression data were normalized to *36B4*. B: Core body temperatures were measured at 10:00 AM under *ad lib* feeding conditions. C: Hematoxylin and eosin staining and Ucp-1 immunostaining in iWAT from CD-wheat and CD-teff mice (left and right columns, respectively). D: Immunofluorescence staining of perilipin (green) in iWAT. E: The size and distribution of adipocytes from iWAT pad of CD-wheat and CD-teff mice quantified by ImageJ. F: Number of adipocyte. n = 3. *p < 0.05, ** *p* < 0.01. n.s. = not significant.

To explore the mechanisms of body weight reduction by teff in the CD-teff group, we analyzed the mRNA levels of thermogenic and beige adipocyte marker genes in the inguinal fat pad. Interestingly, a marked increase in *Ucp-1* and *Cidea* expression was observed in the CD-teff group compared with the CD-wheat group (Fig 6A). However, these beige marker genes were comparable between the HFD-wheat and HFD-teff groups (S3 Fig). Furthermore, a slight but significant increase in body temperature was observed in the CD-teff group (Fig 6B). Histologically, iWAT from CD-teff mice showed high density of hematoxylin and eosin-stained structures along with increased Ucp-1 staining (Fig 6C). Immunofluorescence staining against perilipin, a lipid droplet–specific marker, demonstrated that CD-teff mice had significantly smaller lipid droplets compared with CD-wheat mice (Fig 6D and 6E). Moreover, the number of adipocytes was higher in the CD-teff group than in the CD-wheat group (Fig 6F).

### Teff diet shows better glucose tolerance during a pair feeding study

**Fig 7:**
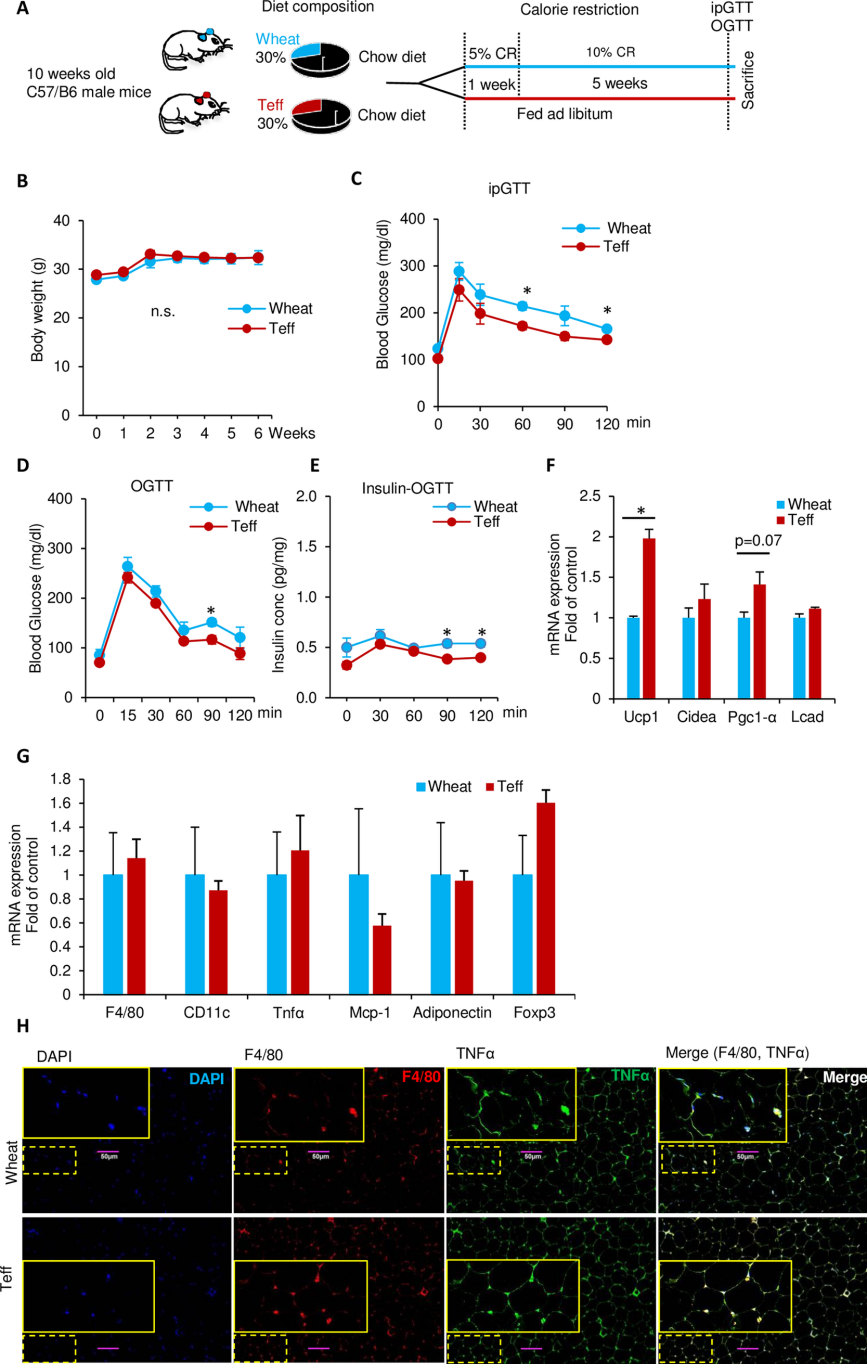
Body weight matching by calorie restriction and glucose tolerance. A: Experimental design. B: Body weight. C: Intraperitoneal glucose tolerance test (IPGTT) at week 6 (2 g/kg). D: Oral glucose tolerance test (OGTT) at week 6 (2 g/kg). E: Insulin concentration during OGTT. F: mRNA levels of thermogenic and beige adipocyte marker genes in the inguinal adipose tissue from mice fed for 6 weeks with CD-wheat or CD-teff. All mRNA expression data were normalized to *36B4*. C: Immunofluorescence staining with DAPI (blue), and with antibodies for F4/80 (red), and TNFα (green) in adipose tissue (scale bar, 100 μm). n = 3–4. *p < 0.05, **. n.s. = not significant.

To justify the role of weight gain/loss in the GTT, we performed pair feeding to match the body weight between the CD-wheat and CD-teff groups. After feeding with 5% CR during the first week and10% CR for 5 weeks (Fig 7A), there was no significant difference in body weight between the two groups (Fig 7B). However, there was a significant difference in IPGTT with a better glucose tolerance in the CD-teff group at 60 and 120 min (*p* < 0.05; Fig 7C), and slightly lower plasma glucose and insulin levels in the CD-teff group in the OGTT (Fig 7D and 7E). Furthermore, the mRNA expression of *Ucp-1*, beige adipocyte marker, was significantly higher in the CD-teff group compared to the CD-wheat group during pair feeding (*p* < 0.05; Fig 7F). Under the weight matching intervention, there was no significant difference in the inflammatory markers between the CD-teff and CD-wheat groups (Fig 7G and 7H). These results indicate that not only weight loss but also beige adipocyte differentiation is the mechanism of improved glucose metabolism by teff.

## Discussion

**Fig 8:**
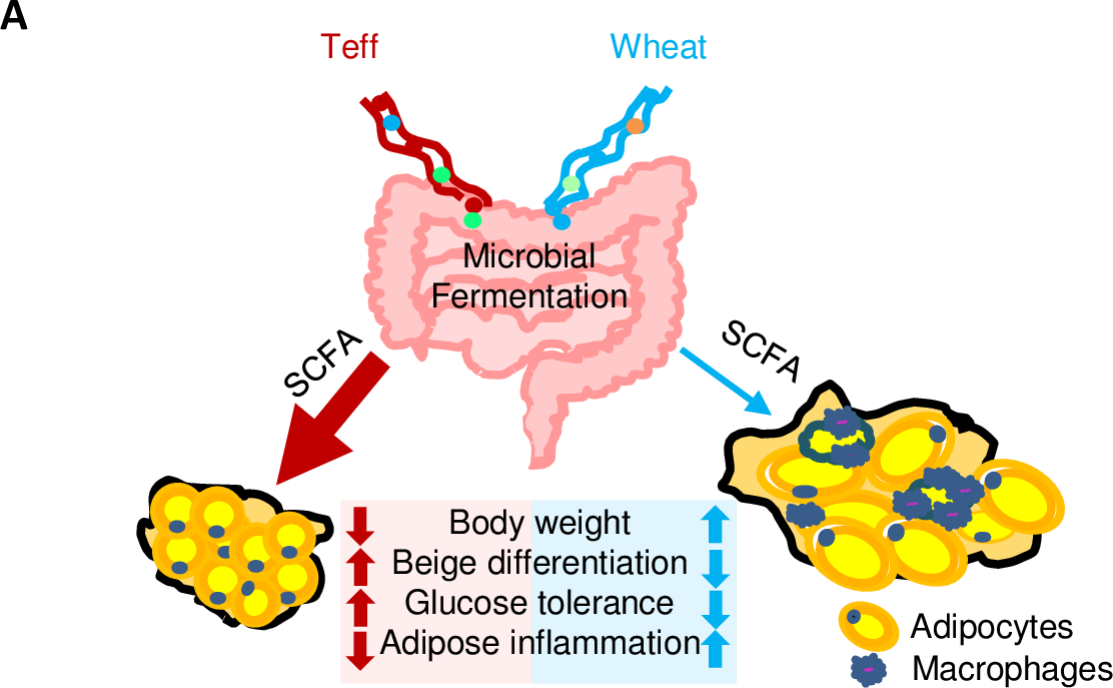
A proposed model of the effects of teff diet on glucose metabolism. Illustrated is a model of how teff improves glucose tolerance by increasing beige adipocyte formation and inhibiting adipose inflammation through increasing SCFAs concentrations.

In this study, we tested the effects of teff on glucose metabolism in mice and found three important results (Fig 8A). First, the teff-containing diet reduced body weight without changing the food intake. Second, the teff-containing diet improved glucose tolerance possibly by enhancing beige adipocyte differentiation. Third, continual teff feeding improved adipose inflammation in the control diet.

The teff-containing diet reduced body weight with no change in food intake. This is consistent with previous observations in epidemiological studies that high dietary fiber consumption is associated with a lower BMI [30]. The World Health Organization has reported that only 2.5% of Ethiopian males and 4.5% of Ethiopian females have a BMI >25 [31]. Although the rate is increasing, the current prevalence of diabetes in Ethiopia is only 3.8% [32]. Injera is a staple food made with teff that may be associated with the maintenance of BMI in Ethiopian people. Notably, immigrants from Ethiopia to Israel exhibited an increased BMI and increased incidence of diabetes, potentially because of changes in food selections [33]. In the current study, the CD-teff diet maintained body weight gain without changes in food intake compared with the CD-wheat diet (Fig 1B). [33]. Our results suggest that this may be explained by enhanced formation of beige adipocytes in the CD-teff group (Fig 6A and 6C–6F). To explorer the mechanism, we focused on the metabolite of dietary fiber, SCFAs. Our results showed that CD-teff has a remarkable potential to generate SCFAs *in vivo* (Fig 5A and 5B) and *in vitro* (Fig 5C–5F), similar to the previous study which whole grain diets increase cecal SCFAs in rats [34]. This is further supported by increases in the villi heights in CD-teff diet (Fig 5G and 5H). The villi heights is increased by direct infusion of SCFAs into cecum [35, 36], which is recognized as a gold standard to monitor intestinal health in animals [37, 38]. Previous study reported that SCFAs stimulated the formation of beige adipocytes through GPR43/41 signaling [39, 40]. Moreover, direct incubation of acetate in 3T3-L1 adipocytes and infusion of acetate in KK-Ay mice clearly showed increased Ucp-1 expression which is the central, hallmark of beige adipocytes [41, 42]. Decreased body weight in CD-teff may be a consequence of increased SCFAs through beige adipocytes formation.

The teff-containing diet improved glucose tolerance in the control diet but not in the HFD. Because this effect was observed during the IPGTT, this phenomenon is not a direct effect of fiber, such as delaying glucose absorption [43]. Rather, body weight reduction may indirectly influence glucose metabolism improved by teff. We speculated that this may be explained in part by body weight differences in the wheat and teff groups. However, the pair feeding experiment showed that body weight reduction by teff is not the sole cause of improved glucose metabolism (Fig 7). Another possible mechanism is adipose inflammation. The CD-teff group showed a dramatic reduction in adipose inflammation markers compared with the CD-wheat group (Fig 3A–3C). The lack of differences in adipose inflammation between the HFD-wheat and HFD-teff groups support this hypothesis (Fig 4A–4C). This finding is consistent with a previous study in which greater consumption of dietary fiber was associated with lower visceral adiposity and multiple biomarkers implicated in inflammation in adolescents [44]. GPR41 and GPR43 are receptors for SCFAs and a recent report has revealed the effect of this pathway by showing that adipose specific GPR43 transgenic mice had reduced numbers of F4/80-positive cells in the adipose tissue [45].

Recently, the role of FoxP3^+^ regulatory T cells (Tregs) was highlighted in inflammatory diseases [46]. Acetate derived from dietary fiber has been reported to upregulate Treg via the action of SCFAs through a GPR43-dependent mechanism or histone deacetylase inhibition [46]. In our study, we hypothesized that there would be increased expression of Foxp3, a transcription factor associated with Tregs, in the adipose tissue, however our data showed decreased Foxp3 expression in the CD-teff group, suggesting acetate had a minor role in inducing Tregs in the adipose tissue.

Teff reduced the postprandial glucose and insulin concentrations both in CD and HFD fed mice examined by the OMTT (Figs 1E and 2E). This suggests that each glucose excursion during dietary intervention may be lower in the CD-teff and HFD-teff groups compared with the CD-wheat and HFD-wheat groups, respectively.

Dietary fiber has been reported to attenuate weight gain, and enhance insulin sensitivity and GLP-1 secretion [47]. Given that teff is consumed as a whole grain, its richness in resistant starches and fiber could elicit similar effects, such as reduced glucose absorption [48], which is shown by better glucose tolerance during weight matching conditions by CR (Fig 7C). In our study, we also found that the active GLP-1 concentration was higher in the CD-teff group compared with the CD-wheat group, but not in the HFDs (S1 Fig). This may also be explained by the cecal SCFAs levels (Fig 5).

The strengths of this study are two-fold. First, to the best of knowledge, this is the first report of the beneficial effects of teff on glucose metabolism and body weight *in vivo*. Second, this study found a mechanism underlying this phenomenon via adipose inflammation and / or beige adipocyte formation. However, there are some limitations to the study. First, the differences in ingredients between teff and wheat are not solely related to dietary fiber. Although wheat and teff are almost isocaloric (Table 1), higher fat and protein contents may influence its effects. A previous report has shown that the levels of iron, calcium, and copper are higher in teff [49]. In our study, teff contained about 10 times higher iron and calcium compared with wheat (Table 1). The plasma iron and calcium concentrations were similar in this study (S2A and S2B Fig) probably because these nutrients are tightly regulated independently of dietary intake. Second, teff has several subtypes. We chose ivory teff for this study because it is one of the most common types in the markets. Differences in type may result in variation in the effects on glucose metabolism. Third, teff is regularly consumed as injera. The cooking process of injera involves several steps, including fermentation. Therefore, any generalizations must be made with caution. Fourth, beige adipocyte markers changed heterogeneously. Increased Ucp-1 expression in CD-teff mice may not be induced through β3-adrenergic pathway which is a major modulator of cold induced beige adipocyte formation.

In conclusion, teff improved glucose tolerance through beige adipocytes formation accompanied with improved adipose inflammation potentially by short chain fatty acids derived from dietary fiber. Further experiments are necessary to elucidate the mechanisms including microbiota and usefulness in clinical situations.

## Supporting information

S1 Fig Active GLP-1 concentration in mice fed with CD-wheat, CD = teff, HFD-wheat, and HFD-teff for 14 weeks. Sample were collected under deep anesthesia after 15 hours of fasting. *p < 0.05, n = 4–5 in each groups.
(TIF)

S2 Fig Plasma concentration of Ca. **IP and Fe in mice fed with CD-wheat, CD-teff, HFD-wheat, and HFD-teff for 9 weeks.** Sample were collected under deep anesthesia after 15 hours of fasting or fed state. *p < 0.05, n = 4 in each groups.
(TIF)

S3 Fig **A: mRNA levels of thermogenic and beige adipocyte marker genes in the inguinal adipose tissue from mice fed for 14 weeks with HFD-what or HFD-teff.** All mRNA expression data were normalized to 36B4.
(TIF)
