# Supplementary figures and images for: Improved glucose metabolism by Eragrostis tef potentially through beige adipocyte formation and attenuating adipose tissue inflammation
Source: PLoS One. 2018 Aug 2;13(8):e0201661. doi: 10.1371/journal.pone.0201661 (PMC6072038; doi:10.1371/journal.pone.0201661)

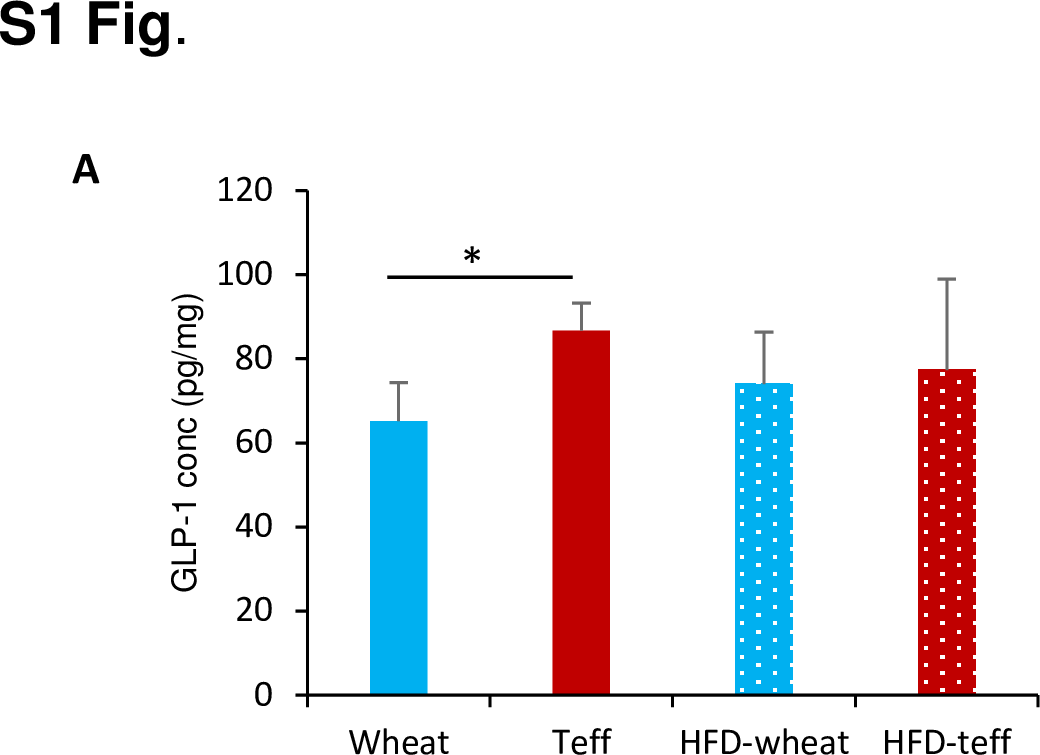

Supplement: S1 Fig — Sample were collected under deep anesthesia after 15 hours of fasting. *p < 0.05, n = 4–5 in each groups. (TIF) [file pone.0201661.s001.tif]

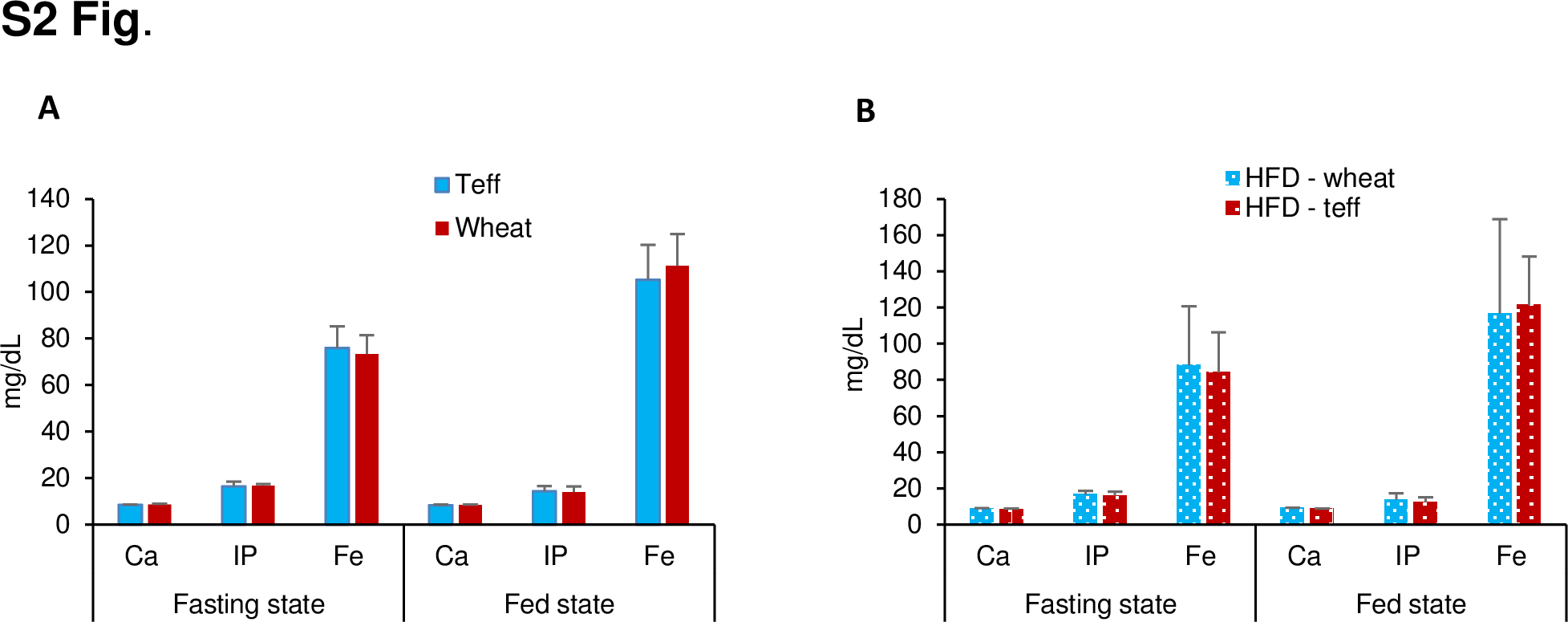

Supplement: S2 Fig — IP and Fe in mice fed with CD-wheat, CD-teff, HFD-wheat, and HFD-teff for 9 weeks. Sample were collected under deep anesthesia after 15 hours of fasting or fed state. *p < 0.05, n = 4 in each groups. (TIF) [file pone.0201661.s002.tif]

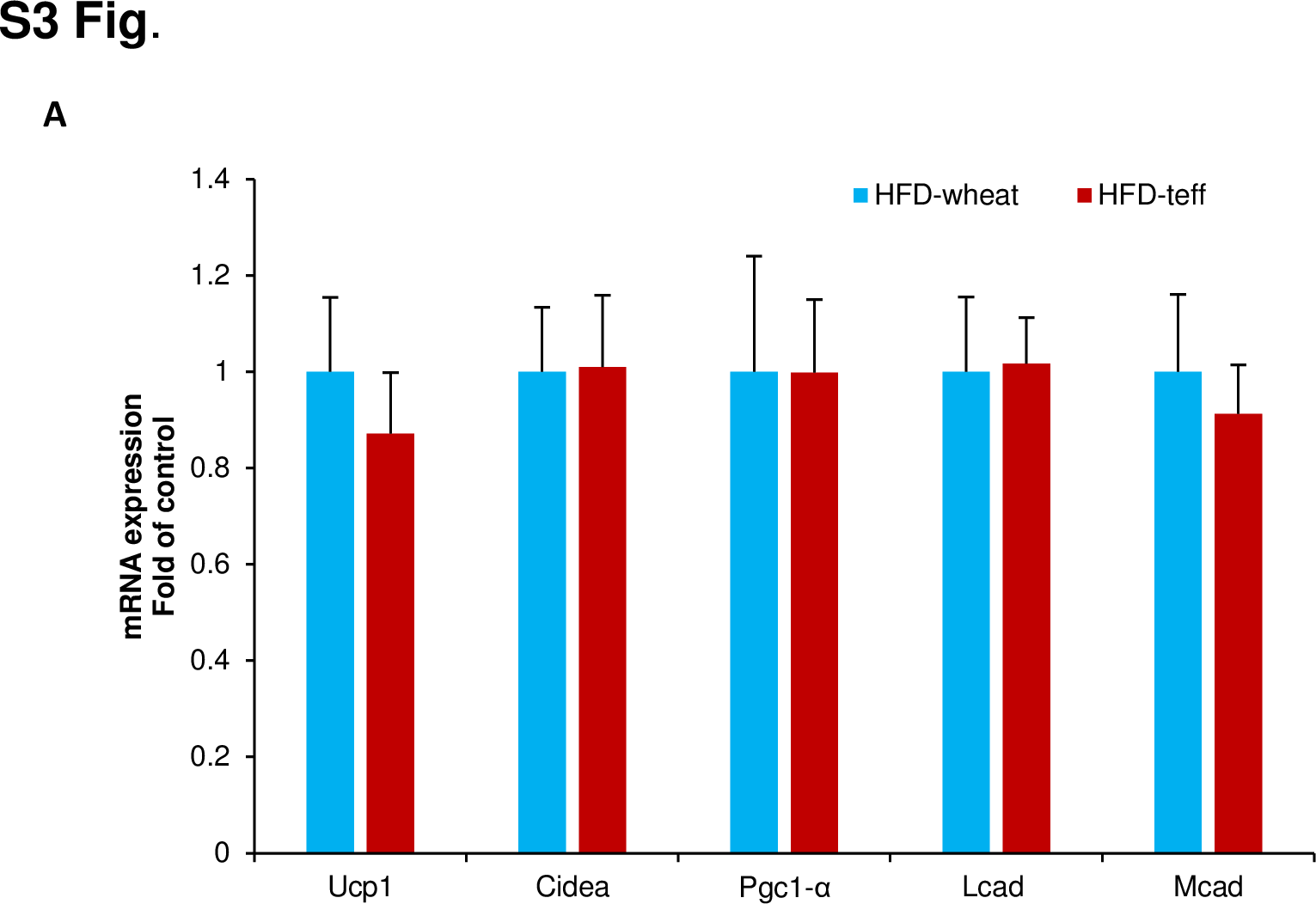

Supplement: S3 Fig — A: mRNA levels of thermogenic and beige adipocyte marker genes in the inguinal adipose tissue from mice fed for 14 weeks with HFD-what or HFD-teff. All mRNA expression data were normalized to 36B4. (TIF) [file pone.0201661.s003.tif]
